# Association of Depression With Past-Month Cannabis Use Among US Adults Aged 20 to 59 Years, 2005 to 2016

**DOI:** 10.1001/jamanetworkopen.2020.13802

**Published:** 2020-08-18

**Authors:** Lauren R. Gorfinkel, Malki Stohl, Deborah Hasin

**Affiliations:** 1 Department of Epidemiology, Mailman School of Public Health, Columbia University, New York, New York; 2 New York State Psychiatric Institute, New York; 3 Department of Psychiatry, Vagelos College of Physicians and Surgeons, Columbia University, New York, New York

## Abstract

**Importance:**

Despite studies showing that repeated cannabis use may worsen depressive symptoms, the popular media increasingly presents cannabis as beneficial to mental health, and many members of the public view cannabis as beneficial for depression. Therefore, cannabis use among individuals with depression may be becoming more prevalent.

**Objective:**

To examine the association of depression with past-month cannabis use among US adults and the time trends for this association from 2005 to 2016.

**Design, Setting, and Participants:**

This repeated cross-sectional study used data from 16 216 adults aged 20 to 59 years who were surveyed by the National Health and Nutrition Examination Survey, a national, annual, cross-sectional survey in the United States, between 2005 and 2016. Data analysis was conducted from January to February 2020.

**Exposures:**

Survey year and depression, as indicated by a score of at least 10 on the Patient Health Questionnaire–9.

**Main Outcomes and Measures:**

Any past-month cannabis use (ie, ≥1 use in the past 30 days) and daily or near-daily past-month cannabis use (ie, ≥20 uses in the past 30 days). Logistic regression was used to examine time trends in the prevalence of cannabis use, depression, and the association between cannabis use and depression from 2005 to 2016.

**Results:**

The final analysis included 16 216 adults, of whom 7768 (weighted percentage, 48.9%) were men, 6809 (weighted percentage, 66.4%) were non-Hispanic White participants, and 9494 (weighted percentage, 65.6%) had at least some college education. They had a weighted mean age of 39.12 (95% CI, 38.23-39.40) years. Individuals with depression had 1.90 (95% CI, 1.62-2.24) times the odds of any past-month cannabis use and 2.29 (95% CI, 1.80-2.92) times the odds of daily or near-daily cannabis use compared with those without depression. The association between cannabis use and depression increased significantly from 2005 to 2016. The odds ratio for depression and any past-month cannabis use increased from 1.46 (95% CI, 1.07-1.99) in 2005 to 2006 to 2.30 (95% CI, 1.82-2.91) in 2015 to 2016. The odds ratio for depression and daily or near-daily past-month cannabis use increased from 1.37 (95% CI, 0.81-2.32) in 2005 to 2006 to 3.16 (95% CI, 2.23-4.48) in 2015 to 2016.

**Conclusions and Relevance:**

The findings of this study indicate that individuals with depression are at increasing risk of cannabis use, with a particularly strong increase in daily or near-daily cannabis use. Clinicians should be aware of these trends and the evidence that cannabis does not treat depression effectively when discussing cannabis use with patients.

## Key Points

**Question:**

Did the association of depression with past-month cannabis use among US adults change from 2005 to 2016?

**Findings:**

In this repeated cross-sectional study of 16 216 adults, those with depression increased their rates of cannabis use significantly faster than those without depression. In 2005 to 2006, individuals with depression had 46% higher odds of any cannabis use and 37% higher odds of near-daily cannabis use, while in 2015 to 2016, individuals with depression had 130% higher odds of any cannabis use and 216% higher odds of daily cannabis use.

**Meaning:**

In this study, an increasing number of adults with depression used cannabis during the study period.

## Teaser

This cross-sectional study examines the association of depression with past-month cannabis use among US adults and the time trends for this association from 2005 to 2016.

## Introduction

Cannabis is among the most widely used psychoactive substances in the United States.^1^ During the past 2 decades, the prevalence of adult cannabis use has steadily increased^2,3,4^; from 2002 to 2017, past-month cannabis use increased by 98%, and the prevalence of daily or near-daily cannabis use increased by 40%.^1^ Simultaneously, the perceived risks associated with cannabis have decreased,^5,6^ perceived availability has increased,^3^ and state marijuana laws have become more permissive, with 34 states now permitting medical marijuana use and 11 states permitting recreational use.^7^ As a result, concern is increasing regarding potential consequences of cannabis use, including cannabis use disorder, vehicle crashes, social impairment, and mental disorders.^8,9,10^

A particular concern is the potential adverse effects of cannabis on major depression, which is consistently associated with cannabis use.^2,11,12,13,14,15,16^ In the United States, major depression is among the most common reasons for seeking health care,^17^ affecting more than 17 million adults and resulting in considerable impairment.^18,19^ Existing evidence indicates that cannabis may worsen depressive symptoms,^20,21,22^ particularly if used regularly.^20,21,23^ However, much of the public views cannabis as helpful for treating depression. In a national survey of US adults, nearly 50% reported their belief that cannabis is beneficial for anxiety or depression, while only 15% believed cannabis increases the risk of these conditions.^24^ Depression is among the most commonly self-reported reasons for cannabis use,^25,26,27,28^ and nearly 25% of adults with mood or anxiety disorders report using cannabis to self-medicate.^29,30^

The belief that cannabis may alleviate symptoms of mood disorders may be partially because of the proliferation of misleading information from media and advertising. Indeed, some of the most popular sources of information about cannabis, including the internet, social media, and the marijuana industry,^31^ have repeatedly been shown to present false or misleading information about the health effects of cannabis.^32,33,34^ Among the most common health claims made in online advertising for recreational cannabis dispensaries is depression treatment.^35^ These messages may be increasing in frequency, while media messaging about marijuana has become more positive over time and includes less information about risks.^34^

Given these changes in cannabis use and media presentation of cannabis as beneficial for health conditions including depression, understanding time trends in the association of cannabis use with depression has become important. Therefore, we investigated the prevalence of any past-month cannabis use and daily or near-daily past-month cannabis use among US adults with and without depression. We then investigated trends in cannabis use and depression over time and trends in the association between any and daily or near-daily cannabis use and depression from 2005 to 2016. We hypothesized that depression would be associated with an increased likelihood of cannabis use (any and daily or near daily) and that this association would have strengthened over the study period.

## Methods

### Setting, Participants, and Procedures

Deidentified data for these analyses come from the National Health and Nutrition Examination Survey (NHANES).^36^ The NHANES is an annual, nationally representative cross-sectional survey of the US civilian population that uses multistage area probability sampling. The stages of sample selection are as follows: (1) selection of primary sampling units (PSUs); (2) segments within PSUs (ie, a block or group of blocks containing a cluster of households); (3) households within segments; and (4) at least 1 participant within each household.^36,37,38^ The 2007 to 2010 NHANES survey cycles oversampled major US demographic subgroups, including Hispanic and non-Hispanic Black persons and low income White persons.^39^ Sample weights and poststratification adjustments during analysis are used to account for oversampling and to control for nonresponse, providing study estimates reflecting US Census Bureau population distributions. Additional information describing the complex sampling weight methodology for the NHANES is presented elsewhere.^36,37,38^

In the first step of the NHANES survey, trained interviewers conducted interviews in participants’ homes. In the second step, participants were asked to attend a mobile examination center (MEC) for physical examinations and additional questionnaires, which generally occurred within 1 to 2 weeks after the in-home interview.^40^ Informed consent was obtained from all participants, and all data collection protocols were approved by the National Center for Health Statistics research ethics review board. The environment, equipment, and specimen collection within MECs were standardized. As compensation for participating, all participants were given a cash payment as well as reimbursement for any transportation and child or elder care.^40^ The proportion of participants who attended the MEC examination following the at-home interview between 2005 to 2006 and 2015 to 2016 was very high, ranging from 95.7% to 97.3%.^41^

Because NHANES data are released in biannual groupings, the current study merged and analyzed a total of 6 data sets: 2005 to 2006, 2007 to 2008, 2009 to 2010, 2011 to 2012, 2013 to 2014, and 2015 to 2016. The 6 data sets were concatenated, with a variable added to indicate survey year. Cannabis use was covered among participants aged 20 to 59 years in all study periods, so the sample for analysis included all NHANES respondents aged 20 to 59 years. Demographic covariates were assessed during the home visit by an interviewer, while depression and cannabis use were assessed using the audio computer-assisted personal self-interview questionnaire in the MEC.^42^ Using this format, respondents hear questions through headphones and read questions on a computer screen. Participants then respond to the questions at their own speed using a touch screen. Because the data are publicly available and deidentified, the current study was exempt from review by the Columbia University Medical Center institutional review board. This report was prepared using the Strengthening the Reporting of Observational Studies in Epidemiology (STROBE) reporting guideline.

### Inclusion and Exclusion Criteria

Pooling NHANES survey data from 2005 to 2016 yielded a sample size of 20 369 adults aged 20 to 59 years. The final analytic sample excluded those who were missing information on cannabis use (444 [2.2%]), depression (44 [0.2%]), both (2157 [10.6%]), or control covariates (1508 [7.4%]). The final sample contained 16 216 US adults. The percentage of nonmissing observations was approximately 80% in each survey year (range, 76.9%-82.4%). To test for potential bias, a post hoc analysis examined time trends and the crude associations of depression with cannabis use in a sample including those missing at least 1 control covariate (17 724 participants), demonstrating similar results to those in the final sample (eTable 1 in the Supplement). Demographic characteristics of participants missing information on probable depression and/or past-month cannabis use are presented in eTable 2 in the Supplement.

### Measures

#### Outcomes

Two measures were created to examine past-month cannabis use, both of which dichotomized responses to the question, “How many times have you used cannabis in the past 30 days?” Any past-month cannabis use was defined as using cannabis at least 1 time vs 0 times in the past 30 days. Daily or near-daily past-month use was defined as using cannabis at least 20 times vs less than 20 times in the past 30 days, consistent with prior studies.^1,43^

#### Main Exposure

Probable depression was identified using the Patient Health Questionnaire–9 (PHQ-9). This is a validated and widely used measure of depression in clinical research.^44^ Consistent with previous research, major depression was dichotomized as absent vs present with scores of less than 10 and at least 10, respectively.^44^

#### Control Covariates

Control covariates included age (20-26, 27-34, 35-42, 43-50, and 51-59 years), gender (men and women), race (non-Hispanic White, non-Hispanic Black, Mexican-American, other Hispanic, and other race or multiracial), education (<high school, high school diploma or GED, and at least some college), marital status (married or living together, previously married, and never married), annual family income (≤$19 999, $20 000-$34 999, $35 000-$74 999, and ≥$75 000), survey year (2005-2006, 2007-2008, 2009-2010, 2011-2012, 2013-2014, and 2015-2016), and past-year alcohol, heroin, and methamphetamine use. Age categories were defined to allow for comparison with cannabis use prevalence among adults aged 26 years or younger and those older than 26 years, which have been reported elsewhere.^1^

### Statistical Analysis

A new sample weight variable for the 6 combined data sets from 2005 to 2016 was created by dividing each MEC sample weight by the total number of data sets, then summing them.^45^ Prevalence for all outcomes and demographic characteristics were calculated for each survey year. Unadjusted and covariate-adjusted logistic regression models were used to assess linear time trends in both cannabis outcomes and depression from 2005 to 2016 and the association between depression and cannabis outcomes, adjusting for survey year. Within the first set of models, unadjusted and adjusted odds ratios (ORs and aORs, respectively) reflected the increased or decreased odds of depression or cannabis use between 2005 and 2016. Within the second set of models, the ORs and aORs reflected the increased or decreased odds of cannabis use by depression status, adjusting for survey year. To determine whether associations differed by survey year, the logistic models were rerun including an interaction term between survey year and depression. In all analyses, Taylor series estimation methods were used to obtain standard error estimates. Statistical significance was set at α < .05 for all analyses, and tests were 2-tailed. All analyses were conducted using SAS version 9.4 (SAS Institute).

To check robustness of results, analyses were rerun using a redefined depression variable to potentially indicate severe major depression. This was dichotomized as a PHQ-9 score of at least 20 vs less than 20.^44^

## Results

The final study sample consisted of 16 216 US adults aged 20 to 59 years, of whom 7768 (weighted percentage, 48.9%) were men, 6809 (weighted percentage, 66.4%) were non-Hispanic White participants, 9494 (weighted percentage, 65.6%) had at least some college education, 11 602 (weighted percentage, 62.4%) had an annual family income of less than $75 000, and 9812 (weighted percentage, 63.5%) were married or living together. Sample characteristics of included participants by survey year are presented in Table 1.

**Table 1.** Characteristics of Included Participants by Survey Year, National Health and Nutrition Examination Survey 2005-2006 to 2015-2016

Variable	Participants, No. (weighted %)						
	Survey year						Total (N = 16 216)
	2005-2006 (n = 2516)	2007-2008 (n = 2624)	2009-2010 (n = 2878)	2011-2012 (n = 2586)	2013-2014 (n = 2893)	2015-2016 (n = 2719)	
Age, y							
20-26	572 (18.0)	472 (18.7)	549 (18.0)	552 (19.7)	557 (20.1)	501 (18.8)	3203 (19.0)
27-34	574 (19.2)	538 (18.9)	582 (19.7)	546 (19.9)	602 (19.6)	581 (19.8)	3423 (19.5)
35-42	497 (21.0)	551 (19.9)	592 (19.8)	502 (17.8)	593 (19.4)	551 (19.3)	3286 (19.5)
43-50	471 (22.0)	548 (22.3)	591 (20.8)	477 (20.5)	579 (19.7)	533 (20.3)	3199 (20.9)
51-59	402 (19.7)	515 (20.1)	564 (21.6)	509 (22.1)	562 (21.2)	553 (21.8)	3105 (21.2)
Men	1137 (48.0)	1264 (48.1)	1414 (50.4)	1290 (49.3)	1386 (49.1)	1277 (48.2)	7768 (48.9)
Race/ethnicity							
Non-Hispanic							
White	1123 (71.8)	1158 (68.8)	1369 (67.7)	970 (66.1)	1205 (63.5)	884 (62.7)	6809 (66.4)
Black	578 (11.4)	561 (11.9)	516 (11.4)	654 (11.6)	578 (11.8)	596 (11.8)	3483 (11.7)
Mexican American	511 (8.0)	513 (9.1)	535 (9.1)	260 (8.1)	397 (10.2)	477 (9.8)	2693 (9.1)
Other Hispanic	90 (3.5)	285 (4.8)	299 (5.3)	242 (6.8)	249 (6.0)	319 (6.5)	1484 (5.6)
Other race or multiracial	114 (5.4)	107 (5.5)	159 (6.5)	460 (7.3)	464 (8.5)	443 (9.1)	1747 (7.2)
Education							
<High school	504 (12.5)	643 (16.5)	649 (15.1)	420 (12.2)	477 (12.4)	471 (11.7)	3164 (13.4)
High school diploma or GED	552 (22.1)	610 (22.6)	678 (22.4)	512 (18.7)	618 (21.0)	588 (19.5)	3558 (21.0)
≥Some college	1460 (65.4)	1371 (60.8)	1551 (62.5)	1654 (69.1)	1798 (66.6)	1660 (68.7)	9494 (65.6)
Annual family income, $							
≤19 999	486 (13.5)	570 (15.8)	688 (16.7)	682 (19.4)	580 (15.1)	511 (13.6)	3517 (15.8)
20 000-34 999	477 (15.6)	557 (16.6)	621 (15.0)	493 (15.8)	568 (16.3)	495 (15.0)	3211 (15.7)
35 000-74 999	869 (35.7)	782 (30.6)	809 (30.8)	700 (29.9)	825 (28.9)	889 (30.8)	4874 (30.9)
≥75 000	684 (35.2)	715 (37.0)	760 (37.5)	711 (34.8)	920 (39.6)	824 (40.6)	4614 (37.6)
Marital status							
Married or living together	1660 (68.4)	1607 (64.6)	1727 (62.9)	1424 (59.5)	1716 (62.0)	1678 (65.2)	9812 (63.5)
Previously married	319 (13.0)	403 (13.0)	444 (14.1)	353 (13.5)	417 (13.3)	372 (12.6)	2308 (13.3)
Never married	537 (18.7)	614 (22.4)	707 (23.0)	809 (26.9)	760 (24.7)	669 (22.2)	4096 (23.2)
Past-year substance use							
Alcohol	1388 (61.4)	1519 (61.8)	1775 (66.4)	1593 (69.2)	1707 (64.3)	1565 (64.7)	9547 (64.8)
Heroin	6 (0.2)	4 (0.2)	11 (0.3)	3 (0.2)	8 (0.2)	8 (0.3)	40 (0.2)
Cocaine	46 (1.6)	64 (1.7)	59 (1.6)	51 (1.9)	40 (1.1)	53 (1.7)	313 (1.6)
Methamphetamine	14 (0.5)	12 (0.4)	3 (0.1)	19 (0.6)	24 (0.6)	20 (0.6)	92 (0.5)
Probable depression^a^	169 (5.6)	265 (7.9)	294 (8.1)	225 (7.5)	239 (7.8)	221 (7.7)	1413 (7.5)

Abbreviation: GED, general education development.

^a^ Defined as Patient Health Questionnaire–9 score at least 10.

### Any Past-Month Cannabis Use From 2005 to 2016

The prevalence of any past-month cannabis use increased from 12.24% (SE, 1.29) in 2005 to 2006 to 17.30% (SE, 1.90) in 2015 to 2016 (Table 2). This overall change was significant (OR, 1.09; 95% CI, 1.03-1.15, *P* < .001), ie, the estimated odds of cannabis use increased by approximately 9% between every 2-year time period.

**Table 2.** Change in Prevalences of Past-Month Cannabis Use Among US Adults Aged 20 to 59 Years, National Health and Nutrition Examination Survey 2005-2006 to 2015-2016

Past-month cannabis use^a^	Prevalence, weighted % (SE)^b^						Change over time, OR (95% CI)
	2005-2006	2007-2008	2009-2010	2011-2012	2013-2014	2015-2016	
Any	12.24 (1.29)	11.82 (0.55)	13.68 (0.99)	14.19 (1.11)	14.75 (0.78)	17.30 (1.90)	1.09 (1.03-1.15)
Daily or near-daily^c^	3.78 (0.61)	3.29 (0.44)	4.98 (0.54)	4.42 (0.53)	5.46 (0.32)	6.06 (1.03)	1.12 (1.04-1.21)

Abbreviation: OR, odds ratio.

^a^ Control covariates were gender, age, race, education, marital status, annual family income.

^b^ Total number of participants was 16 216.

^c^ Using cannabis at least 20 times in the past month.

### Daily or Near-Daily Past-Month Cannabis Use From 2005 to 2016

The prevalence of daily or near-daily past-month cannabis use increased from 3.78% (SE, 0.61) in 2005 to 2006 to 6.06% (SE, 1.03) in 2015 to 2016 (Table 2). This change was also significant (OR, 1.12; 95% CI, 1.04-1.21, *P* < .001), with the estimated odds of daily or near-daily use increasing by approximately 12% between every 2-year period.

### Depression From 2005 to 2016

Comparing consecutive survey years (eg, 2005-2006 vs 2007-2008, 2007-2008 vs 2009-2010), none of the changes in the odds of depression were significant. Odds ratios for individual comparisons between survey years are presented in eTable 3 in the Supplement. Among the 1413 participants who screened positive for major depression, 1110 (weighted percentage, 80.9%) endorsed at least some resulting difficulty in their work, home or relationships.

### Depression and Any Past-Month Cannabis Use

Overall, the crude OR indicating the association of depression with any past-month cannabis use was 2.03 (95% CI, 1.74-2.36, *P* < .001), while the aOR was 1.90 (95% CI, 1.62-2.12; *P* < .001). The observed prevalence of past-month cannabis use by depression status during the study period are presented in Table 3. Consistently, individuals with probable depression had an elevated prevalence of past-month use (eg, 2005-2006, individuals with vs without depression, 17.81% [SE, 3.77] vs 11.90% [SE, 1.18]; 2015-2016, 31.88% [SE, 2.89] vs 16.09% [SE, 1.90]).

**Table 3.** Change in the Prevalence of Past-Month Cannabis Use by Depression Status and Association Between Depression and Past-Month Cannabis Use Among US Adults Aged 20 to 59 Years, National Health and Nutrition Examination Survey 2005-2006 to 2015-2016

Past month cannabis use	Prevalence, weighted % (SE)^a^						Association between past-month cannabis use and depression, OR (95% CI)	
	2005-2006	2007-2008	2009-2010	2011-2012	2013-2014	2015-2016	Crude	Adjusted^b^
Any								
No probable depression	11.90 (1.18)	11.32 (0.60)	12.99 (0.96)	13.36 (0.94)	13.79 (0.71)	16.09 (1.90)	1 [Reference]	1 [Reference]
Probable depression	17.81 (3.77)	17.68 (2.19)	21.51 (3.31)	24.36 (4.58)	26.09 (3.53)	31.88 (2.89)	2.03 (1.74-2.36)	1.90 (1.62-2.24)
Daily or near-daily								
No probable depression	3.59 (0.57)	3.22 (0.45)	4.74 (0.55)	3.88 (0.49)	4.95 (0.43)	5.27 (0.92)	1 [Reference]	1 [Reference]
Probable depression	7.03 (2.84)	4.00 (1.26)	7.78 (1.49)	11.05 (3.17)	11.47 (2.13)	15.59 (3.19)	2.39 (1.88-3.04)	2.29 (1.80-2.92)

Abbreviation: OR, odds ratio.

^a^ Total number of participants was 16 216.

^b^ Control covariates included gender, age, race, education, marital status, annual family income, and past-year alcohol, cocaine, heroin, and methamphetamine use.

### Depression and Daily or Near-Daily Past-Month Cannabis Use

The crude odds ratio for depression and daily or near-daily past-month use was 2.39 (95% CI, 1.88-3.04; *P* < .001), while the aOR was 2.29 (95% CI, 1.80-2.92; *P* < .001). The prevalence of daily or near-daily past-month cannabis use by depression status during the study period are presented in Table 3. Individuals with probable depression had consistently higher prevalences of past-month daily or near-daily cannabis use than individuals without depression (eg, 2005-2006, individuals with vs without depression, 7.03% [SE, 2.84] vs 3.59% [SE, 0.57]; 2015-2016, 15.59% [SE, 3.19] vs 5.27% [SE, 0.92]).

### Change in the Association Between Depression and Cannabis Use From 2005 to 2016

The interaction results of depression and survey year indicated that the associations between depression and any cannabis use as well as daily or near-daily cannabis use increased significantly between 2005 to 2006 and 2015 to 2016 (any use: χ^2^_1_ = 4.35; *P* = .04; daily or near-daily use: χ^2^_1_ = 5.34; *P* = .02). Overall, the OR for depression and any past-month cannabis use increased from 1.46 (95% CI, 1.07-1.99) in 2005 to 2006 to 2.30 (95% CI, 1.82-2.91) in 2015 to 2016. The OR for depression and daily or near-daily past-month cannabis use increased from 1.37 (95% CI, 0.81-2.32) in 2005 to 2006 to 3.16 (95% CI, 2.23-4.48) in 2015 to 2016.

### Sensitivity Analyses

After redefining the primary exposure variable as positive if the PHQ-9 score potentially indicated severe major depression (ie, score ≥20), results were virtually unchanged. The aOR indicating the association between any past-month cannabis use and severe depression was 2.22 (95% CI, 1.32-3.74; *P* = .003), while the aOR for daily or near-daily past-month use and severe depression was 3.26 (95% CI, 1.62-6.57; *P* = .001). The tests for interaction between severe depression and survey year were not significant for the association between severe depression and any cannabis use (χ^2^_1_ = 3.84; *P* = .05) but showed a significant increase in the association between severe depression and daily or near-daily cannabis use over time (χ^2^_1_ = 7.71; *P* = .006).

## Discussion

This study used nationally representative data to examine trends in the association between depression and cannabis use in US adults aged 20 to 59 years using the NHANES. Overall, there were 3 major findings, as follows: (1) the prevalence of any past-month cannabis use and daily or near-daily cannabis use increased from 2005 to 2016, while the prevalence of depression remained stable; (2) individuals with depression had approximately double the odds of using cannabis compared with people without depression; and (3) the association between depression and cannabis use strengthened from 2005 to 2016.

The prevalence of adult cannabis use increased from 2005 to 2016. In 2005 to 2006, the prevalence of any past-month cannabis use was 12.2%, and the prevalence of daily or near-daily past-month cannabis use was 3.8%. By 2015 to 2016, these figures had increased to 17.3% and 6.1%, respectively. These trends are consistent with findings from other epidemiologic studies of cannabis use.^1^ Therefore, the current study provides an additional source of evidence for increasing cannabis use among US adults during the past decade. Given that cannabis use, particularly heavy or frequent use, is associated with some harmful outcomes (such as vehicle crashes, impaired memory, and psychiatric symptoms),^8,9,10^ these results reinforce the importance of monitoring cannabis use and mitigating its consequences.

Second, consistent with our hypotheses and prior research,^15,46^ we observed a significant association between any past-month cannabis use and depression. We also observed a stronger association between depression and daily cannabis use (aOR, 2.29; 95% CI, 1.80-2.92) compared with depression and any cannabis use (aOR, 1.90; 95% CI, 1.62-2.24). In 2015 to 2016, more than 30% of individuals with depression reported using cannabis in the past 30 days, and more than 15% reported using cannabis approximately daily. Although the current study is cross-sectional and therefore cannot provide information on the direction of the association between depression and cannabis use, these findings are concerning, given that both heavier use and depression have been associated with increased risk of cannabis-related harm.^8,47,48^ Individuals with depression who use cannabis may represent a high-risk group for cannabis-involved adverse consequences, potential worsening of depressive symptoms, and suicidality.^49^ In health care settings where depression is addressed, discussion with patients regarding the frequency of their cannabis use, strategies for cutting back, and the effects of cannabis on depressive symptoms may be useful.

Finally, we found a marked increase in the association between any and daily or near-daily cannabis use and depression from 2005 to 2016. In 2005 to 2006, individuals with depression had 46% higher odds of any cannabis use and 30% higher odds of near-daily cannabis use. In 2015 to 2016, individuals with depression had 130% higher odds of any cannabis use and 216% higher odds of daily cannabis use. These results suggest that over time, a higher proportion of individuals with depression are using cannabis. This could be the case if an increasing number of individuals with depression are using cannabis to self-medicate, potentially influenced by media and advertising presenting cannabis as beneficial to health.^32,33,34,35^ These results could also be interpreted as indicating that an increasing proportion of individuals who use cannabis are developing depression. However, if this were true, we would expect to see that as the prevalence of cannabis use increased, the prevalence of depression increased as well. However, depression stayed relatively stable during the study period, not supporting the latter interpretation.

These findings are also consistent with a 2020 article using data from the National Survey on Drug Use and Health (NSDUH).^50^ Although the NSDUH is a common source of information on substance use in the US, other sources are needed to confirm this survey’s findings. In presenting information from a different nationally representative data set, the NHANES, the current study ensures that these results are not because of idiosyncrasies of the NSDUH methodology and provides valuable information on the growing association between cannabis use and major depression. Moreover, compared with this prior study, we controlled for additional potential confounding covariates, such as other substance use. The current study also found a larger association between depression and any cannabis use and depression and daily cannabis use in the most recent time period (ie, 2015-2016). These findings may suggest that cannabis use among people with depression is increasing at a faster rate and, subsequently, have a current association of greater magnitude than has been previously reported. Future studies should examine this association using other data sets, presenting time-stratified ORs to further confirm these findings.

### Limitations and Strengths

The results of this study should be considered in light of some limitations. First, because past-month cannabis use was self-reported, participants may have underestimated their cannabis use. However, the consistency of our findings with previous research as well as the anonymity of drug use questionnaires in the NHANES surveys suggest that this is unlikely. Second, owing to the cross-sectional nature of this study, the direction of the relationship between depression and cannabis use cannot be known. Probable depression was assessed for the past 2 weeks, while cannabis use was assessed for the past 30 days. It is therefore unclear whether depression existed prior to the 2-week period or whether cannabis use followed depression onset. Nevertheless, our findings of different time trends in depression and cannabis use do not support the hypothesis that a higher proportion of individuals who use cannabis are developing depression. These associations should be further examined in longitudinal studies using more finely grained repeated measures of depression and cannabis use and also potential sources of participant information on cannabis use (eg, advertising, social media). Third, we examined adults aged 20 to 59 years in 2-year groupings. Future studies should include participants aged 18 to 19 years and 60 years and older and examine annual prevalences, data permitting. Fourth, owing to insufficient information regarding past-year or past-month cigarette use (the time frames of the cannabis and depression variables), we were unable to control for cigarette use. Future research should measure tobacco use in the same time frames as depression and cannabis and include tobacco as an additional control covariate. Finally, the metric for depression, the PHQ-9, is a screening scale rather than a diagnostic instrument. Future research should aim to identify depression using a clinical diagnosis.

The current study also has multiple strengths that warrant mention. Data are nationally representative and used clustered, stratified sampling to ensure representativeness of estimates. The data therefore allowed for the valid measurement of prevalence, time trends, and the association between cannabis and depression among US adults over time, a unique advantage to national survey data. Moreover, despite not having a formal diagnosis, the current study used an extensively validated screening scale for major depression,^5^ which has been used widely in research studies and is simple and short enough (9 questions) for use in large national surveys.

## Conclusions

In this study, we examined the association between depression and cannabis use in US adults aged 20 to 59 years from 2005 through 2016. We observed a significant association between depression and any past-month cannabis use, which strengthened when looking at depression and daily past-month cannabis use. From 2005 to 2016, cannabis use increased in the general population. However, there was a particular increase in cannabis use among people with depression, with approximately 30% of individuals with depression using any cannabis and 15% using cannabis near-daily from 2015 to 2016. While further research to understand the mechanisms underlying the increasingly strong association of depression and frequent cannabis use is needed, the study findings highlight a current need for information campaigns around cannabis and depressive disorders. In addition, clinicians should be aware of the changing trends and the association between cannabis use and depressive symptoms when treating patients. This is particularly important in reference to frequent cannabis use, which is associated with greater risk of harm and potential worsening of depressive symptoms.

## Notes

Supplement. **eTable 1. **Crude Model Results Including Participants Excluded Because of Missing at Least 1 Covariate **eTable 2. **Demographic Characteristics Among Participants Excluded Because of Missing Information on Probable Depression and/or Past-Month Cannabis Use **eTable 3. **Change Over Time in Odds of Probable Depression Among US Adults Aged 20 to 59 Years, National Health and Nutrition Survey 2005-2006 to 2015-2016
