# Supplementary material for: Association of Depression With Past-Month Cannabis Use Among US Adults Aged 20 to 59 Years, 2005 to 2016
Source: JAMA Netw Open. 2020 Aug 18;3(8):e2013802. doi: 10.1001/jamanetworkopen.2020.13802 (PMC7435337; doi:10.1001/jamanetworkopen.2020.13802)
Supplement: Supplement. — eTable 1. Crude Model Results Including Participants Excluded Because of Missing at Least 1 Covariate eTable 2. Demographic Characteristics Among Participants Excluded Because of Missing Information on Probable Depression and/or Past-Month Cannabis Use eTable 3. Change Over Time in Odds of Probable Depression Among US Adults Aged 20 to 59 Years, National Health and Nutrition Survey 2005-2006 to 2015-2016 [file jamanetwopen-3-e2013802-s001.pdf]

Supplementary Online Content

Gorfinkel LR, Stohl M, Hasin D. Association of depression with past-month cannabis use among US adults aged 20 to 59 years, 2005 to 2016. *JAMA Netw Open*. 2020;3(8):e2013802. doi:10.1001/jamanetworkopen.2020.13802

eTable 1. Crude Model Results Including Participants Excluded Because of Missing at Least 1 Covariate
eTable 2. Demographic Characteristics Among Participants Excluded Because of Missing Information on Probable Depression and/or Past-Month Cannabis Use
eTable 3. Change Over Time in Odds of Probable Depression Among US Adults Aged 20 to 59 Years, National Health and Nutrition Survey 2005-2006 to 2015-2016

This supplementary material has been provided by the authors to give readers additional information about their work.

© 2020 Gorfinkel LR et al. *JAMA Network Open*.

**eTable 1.** Crude Model Results Including Participants Excluded Because of Missing at Least 1 Covariate (N=17,768)	
**Association**	**Odds Ratio (95% CI)**
Any past-month cannabis use by probable depression[a]	1.96 (1.70–2.27)
Daily/near-daily past-month cannabis use by probable depression[a]	2.37 (1.86–3.02)
Any past-month cannabis use by survey year	1.08 (1.02–1.15)
Daily/near-daily past-month cannabis use by survey year	1.11 (1.02–1.21
Probable depression[a] by survey year	
2007-2008 vs. 2005-2006	1.48 (1.09–1.99)
2009-2010 vs. 2007-2008	1.03 (0.80–1.33)
2011-2012 vs. 2009-2010	1.00 (0.77–1.29)
2013-2014 vs. 2011-2012	0.97 (0.75–1.26)
2015-2016 vs. 2013-2014	0.88 (0.72–1.09)
[a] As indicated by Patient Health Questionnaire 9 score ≥ 10	

© 2020 Gorfinkel LR et al. *JAMA Network Open*.

eTable 2.
Demographic Characteristics Among Participants Excluded Because of Missing Information on Probable Depression and/or Past-Month Cannabis Use (N=2,157)

Characteristic	Freq (%)
**Survey**	
2005-2006	273 (12.7)
2007-2008	286 (13.3))
2009-2010	436 (20.2)
2011-2012	466 (21.6)
2013-2014	330 (15.3)
2015-2016	366 (17.0)
**Age**	
20-26	356 (16.5)
27-34	485 (22.5)
35-42	506 (23.5)
43-50	433 (20.1)
50-59	377 (17.5)
**Gender (male)**	865 (40.1)
**Race**	
Non-Hispanic white	637 (29.5)
Non-Hispanic black	562 (26.1)
Mexican-American	339 (15.7)
Other Hispanic	227 (10.5)
Other race / Multiracial	392 (18.2)
**Education**	
< HS	600 (27.8)
High School grad or GED	473 (21.9)
At least some college	1,084 (50.3)
**Annual family income**	
$0 - $19,999	580 (14.2)
$20 - $34,999	479 (22.2)
$35 - $74,999	567 (26.3)
≥ $75,000	531 (24.6)
**Marital status**	
Married or living together	1,320 (61.2)
Previously married	310 (14.4)
Never married	527 (24.4)

[a] As indicated by Patient Health Questionnaire 9 score ≥ 10

© 2020 Gorfinkel LR et al. *JAMA Network Open*.

eTable 3. Change Over Time in Odds of Probable Depression Among US Adults Aged 20 to 59 Years, National Health and Nutrition Survey 2005-2006 to 2015-2016

Comparison Between Survey Years	Odds Ratio (95% CI)
2007-2008 vs. 2005-2006	1.44 (1.02–2.02)
2009-2010 vs. 2007-2008	1.03 (0.78–1.34)
2011-2012 vs. 2009-2010	0.93 (0.69–1.26)
2013-2014 vs. 2011-2012	1.04 (0.76–1.41)
2015-2016 vs. 2013-2014	0.98 (0.79–1.23)

[a] As indicated by Patient Health Questionnaire 9 score ≥ 10

© 2020 Gorfinkel LR et al. *JAMA Network Open*.
